# Release and Recharge of Fluoride Ions from Acrylic Resin Modified with Bioactive Glass

**DOI:** 10.3390/polym13071054

**Published:** 2021-03-27

**Authors:** Zbigniew Raszewski, Danuta Nowakowska, Wlodzimierz Wieckiewicz, Agnieszka Nowakowska-Toporowska

**Affiliations:** 1 Spofa Dental Kerr Company, 506-01 Jičin, Czech Republic; Zbigniew.Raszewski@kavokerr.com; 2 Department of Prosthodontics, Wroclaw Medical University, 50-425 Wroclaw, Poland; danuta.nowakowska@umed.wroc.pl (D.N.); protetyka.stom@umed.wroc.pl (W.W.)

**Keywords:** PMMA, bioactive acrylic resin, glass filler, fluoride release, fluoride recharge

## Abstract

Background: Oral hygiene is essential for maintaining residual dentition of partial denture wearers. The dental material should positively affect the oral environment. Fluoride-releasing dental materials help to inhibit microbial colonization and formation of plaque as well as to initiate the remineralization process in the early cavity area. Aim: To evaluate fluoride ion release and recharge capacity, sorption, and solubility of polymethyl methacrylate (PMMA) dental resin modified with bioactive glass addition. Materials and methods: Two bioactive glass materials (5 wt% Kavitan, 10 wt% Kavitan, and 10 wt% Fritex) and pure 10 wt% NaF were added to dental acrylic resin. After polymerization of the modified resins, the release levels of fluoride anions were measured based on color complex formation by using a spectrophotometer after 7, 14, 28, and 35 days of storage in distilled water at 37 °C. Subsequently, specimens were brushed with a fluoride-containing tooth paste on each side for 30 s, and the fluoride recharge and release potential was investigated after 1, 7, and 14 days. Sorption and solubility after 7 days of storage in distilled water was also investigated. Results: The acrylic resins with addition of 10% bioactive glass materials released fluoride ions for over 4 weeks (from 0.14 to 2.27 µg/cm^2^). The amount of fluoride ions released from the PMMA resin with addition of 10 wt% Fritex glass was higher than that from the resin with addition of 10 wt% Kavitan. The acrylic resin containing 10 wt% NaF released a high amount of ions over a period of 1 week (1.58 µg/cm^2^), but the amount of released ions decreased rapidly after 14 days of storage. For specimens containing 5 wt% Kavitan glass, the ion-releasing capacity also lasted only for 14 days. Fluoride ion rechargeable properties were observed for the PMMA resin modified with addition of 10 wt% Fritex glass. The ion release levels after recharge ranged from 0.32 to 0.48 µg/cm^2^. Sorption values ranged from 10.23 μm/mm^3^ for unmodified PMMA resin to 12.11 μm/mm^3^ for specimens modified with 10 wt% Kavitan glass. No significant differences were found regarding solubility levels after 7 days. Conclusions: The addition of 10 wt% Fritex and 10 wt% Kavitan bioactive glass materials to heat-cured acrylic resin may improve its material properties, with bioactive fluoride ion release ability lasting for over 4 weeks. The resin modified with 10 wt% Fritex glass could absorb fluoride ions from the toothpaste solution and then effectively release them. Addition of fluoride releasing fillers have a small effect on sorption and solubility increase of the modified PMMA resin. Clinical significance: The addition of bioactive glass may be promising in the development of the novel bioactive heat-cured denture base resin.

## 1. Introduction

Colonization of the oral cavity by bacteria and formation of dental and denture plaque result in the initiation of caries lesions [1]. Caries is particularly problematic for patients wearing partial removable dentures. The denture base covers the oral mucosa and a large part of the teeth surface, thereby making it difficult to maintain proper oral hygiene of these areas, which reduces saliva flow and facilitates plaque build-up and demineralization of the remaining dentition [2,3]. Following a proper hygiene regimen is crucial for maintaining the remaining dentition and supporting periodontal tissues in healthy condition. One of the most popular caries prevention methods is fluoride delivery. Fluoride reacts with calcium and phosphate ions and forms fluorinated hydroxyapatite and fluoroapatite in the enamel structure, which are more resistant to demineralization [4]. Various preventive products act as fluoride ions donors. Fluoride varnishes, gels, tooth mousses, tooth pastes, and oral solutions of different fluoride concentration are used [5]. The study of Byeon et al. showed, that their fluoride ions release capacity is limited [6]. Fluoride releasing dental restorative materials are a group of filling materials with cariostatic properties. It includes glass–ionomer cements (GIC), giomers, cermets, compomers, and composite materials with differential fluoride releasing ability [7]. The newly designed dental materials should have appropriate mechanical properties, should be biocompatible with oral cavity environment, and should have a long service time [8]. Polymethyl methacrylate (PMMA) is a material commonly used in dentistry. It has vast applications in prosthodontics and orthodontics for fabrication of denture bases, artificial denture teeth, occlusal splints, orthodontic devices, and retainers [9]. A new demand for bioactive dental materials is emerging. These materials should positively affect the oral tissues and should be a source of fluoride, calcium, or phosphate ions, which can initiate the remineralization process in the early caries area [10,11]. Additionally, fluoride anions presence in the oral cavity have antibacterial and antifungal action [12,13]. Various attempts have been made to improve dental materials, such as composite filling materials, fissure sealants, bonding agents and acrylic resins, with fluoride releasing properties. Composite materials were modified by adding sodium fluoride or other fluoride salts to the polymer matrix. In vitro studies revealed that in a short period of time, the fluoride anions were released from the matrix and that the material could act as a donor of fluoride ions. However, it was not possible to maintain the ion releasing effect over a longer period of time [14,15]. Promising results were obtained with incorporation of nanoparticles of calcium fluoride (nCaF_2_) to the nanocomposite filling material [16]. Another efficient source of fluoride ions used in the modification of dental materials are glasses or ceramics, which are a part of glass-ionomer cements (GIC): Full pre-reacted glass–ionomer fillers (F-PRG) and surface pre-reacted glass-ionomer cements (S-PRG) [17,18]. S-PRG fillers have ability of ion release, which promotes remineralization of teeth structures and may neutralize the acidic conditions produced by oral cariogenic microorganisms [19]. They can also reabsorb fluoride ions into the glass matrix from saliva or from direct contact with fluoride-containing toothpastes, mouth rinses, and gels [20,21,22]. This process may fluctuate over an extended period of time [17,23]. In an in vitro study, Mukai et al. showed the demineralization inhibiting capability of bovine teeth dentine in direct contact with S-PRG-modified PMMA, which is a promising result for caries prevention of residual dentition [24]. The ion release levels from PMMA are directly proportional to the filler concentration. However, with the increase in the filler concentration, the flexural strength decreases. Kamaijo et al. reported that the use of less than 20 wt% of the filler is recommended to obtain denture material with adequate mechanical properties [25]. Al-Bakri et al. recommended that the filler percentage should not exceed 10 wt% [26]. Additionally, proportionally with the increase of fluoridated filler content in the material composition the surface roughness of the modified PMMA resin increases [27,28]. Tamura et al. reported that ions released from S-PRG filler induce oxidative stress in Candida albicans which results with diminished growth and biofilm formation, lowering of fungal adhesion capability, inhibiting conversion from the yeast form to the hyphal form, and proteinase production. Therefore, their incorporation in the PMMA resin may result in improved candidiasis control, which is a common problem of denture wearers especially in the elderly group of patients [29].

There are few available studies describing the possible modification of PMMA resin with fluorided glass fillers [24,25,26]. The studies comparing fluoride ion release and recharge capabilities from PMMA modified with bioactive glass fillers of different composition are lacking.

In the present study, two bioactive glasses from GIC and sodium fluoride were tested as additives to heat-cured PMMA resin to determine the dynamics of fluoride release and recharge. The null hypothesis assumed for this study is that there are no differences in fluoride ion release and recharge from the conventional heat-polymerized PMMA and acrylic resins modified with 10 wt% NaF or two different types bioactive glasses.

## 2. Materials and Methods

### 2.1. Fluoride Ion Release

Acrylic resin Superacryl Plus (SpofaDental, Jičín, Czech Republic) was used and modified with two types of bioactive glass materials: Kavitan Plus powder (Pentron, Brea, California, USA) and Fritex (SpofaDental, Jičín, Czech Republic) (Table 1) and sodium fluoride (Sigma-Aldrich, Saint Louis, MO, USA). These fluoride-containing powders were added to the PMMA powder by using a ball mill (Jezirska Porcelana, Czech Republic). The obtained material was then mixed for 2 h (100 g of resin plus 100 g of 10 mm diameter porcelain balls). The prepared powders were then mixed in the proportion of 2.4 g of powder and 1 g of Superacryl Plus monomer. After achieving the dough phase, the materials were polymerized in metal molds of 10 mm diameter and 1 mm thickness. A total of 50 test disc specimens were prepared (Table 2). The unmodified material Superacryl Plus was used as the control group. Each specimen was stored separately in 10 cm^3^ of distilled water at 37 °C.

The concentration of fluoride ions was determined using the spectrophotometric method described by Unal et al. [30]. This method is based on the formation of a colored complex between Fe^3+^ ions (FeCl_3_, Sigma-Aldrich, Saint Louis, MO, USA) and indole-3-acetic acid (Sigma-Aldrich, Saint Louis, MO, USA) in an acidic environment. The absorption of the colored pink complex was estimated using a Helios spectrophotometer (Thermo Fisher Scientific, Waltham, MA, USA) at the wavelength of 525 nm.

**Figure 1:**
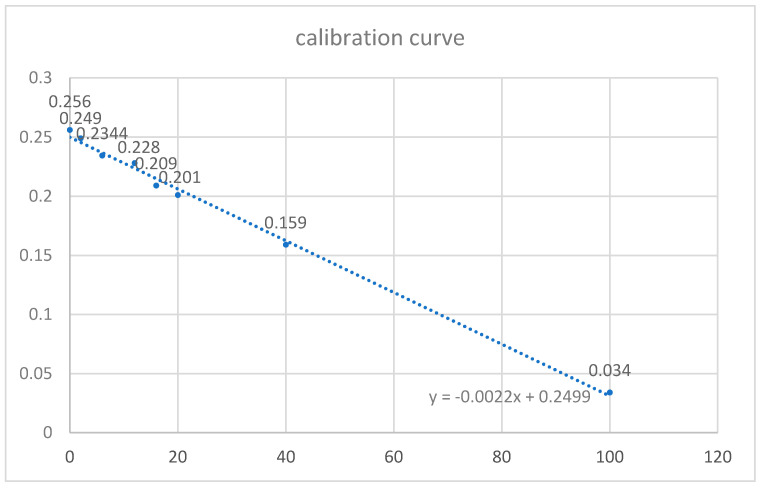
Calibration curve for fluoride ion concentration based on color complex formation between iron ions and indole-3-acetic acid.

Initially, a standard calibration curve was prepared with known concentrations of sodium fluoride: 0, 2, 6, 12, 16, 20, 40, and 100 ppm. A solution of NaF (Sigma-Aldrich, Saint Louis, MO, USA) with 1000 ppm concentration was prepared in a volumetric flask. A defined amount of fluid was then taken from this solution and added to other volumetric flasks to obtain the appropriate concentrations of fluoride. To measure the absorbance, 1 cm^3^ of appropriate solutions of NaF was taken from volumetric flasks, to which 2 cm^3^ of 0.01 mol FeCl_3_ solution and 1 cm^3^ of 0.01 mol indole-3-acetic acid were added. Next, 1 drop of 12.5% HCl was added to this solution to provide an acidic environment of pH ranging from 1.5–2. The entire mixture was supplemented with distilled water to a volume of 5 cm^3^. The prepared solutions were then heated in a water bath at 60 °C for 10 min to achieve color complex of the maximum absorbance. Following the heat treatment, a pink color appeared in each solution, the intensity of which was inversely proportional to the concentration of fluoride ions. A standard curve (Figure 1) was generated by plotting the concentration of the solutions (X-axis) against absorbance values (Y-axis). Next, 1 cm^3^ of distilled water in which each specimen was stored was taken. The process was repeated to achieve the pink color complex. The absorbance was measured using the spectrophotometer to evaluate the amount of released fluoride ions. This amount was multiplied by 10 because of the total volume of storage medium for each specimen and divided by specimen surface in cm^2^.

### 2.2. Recharge of Fluoride Ions

Acrylic specimens were brushed with a toothbrush (Oral-B Pro-Expert All-In-One, Procter & Gamble Company, Cincinnati, OH, USA) and a toothpaste containing 1450 ppm of fluoride ions (Colodent, Colgate Palmolive, NY, USA) on each side for 30 s. The acrylic discs with the applied toothpaste were left in distilled water for 10 h. This process was considered to simulate the storage of the denture by patient in water at nighttime. The specimens were washed under running water, and 10 cm^3^ of distilled water was again added to each of them. After 24 h of immersion, the amount of released fluoride ions was again determined using the spectrophotometric method. The test was repeated after 7 and 14 days.

### 2.3. Sorption and Solubility

Specimens of diameter 50 mm and thickness of 1 mm according to ISO 20795-1:2013 [31] were cured with acrylic resins and mixtures of acrylic resins and different bioactive glass and sodium fluoride. For each composition 5 samples were polymerized (totally 25 discs). Specimens were dried in dessicator with use of silica gel (Sigma-Aldrich, Saint Louis, MO, USA) and weighed with use of analytical balance (Precioza 256) until constant mass was established. This value was noted as initial weight (W1). Afterwards, specimens were placed in distilled water for 7 days at 37 °C. After 7 days of immersion, specimens were removed from the distilled water, washed and dried with paper, and weighed. This value was noted as mass after absorption [W_2_]. Specimens were then dried again in a desiccator over silica gel until constant weight was obtained [W_3_].

Sorption and solubility values were calculated using the formula:

(1) $$\mathrm{Sorption}\left[\frac{\mu g}{{\mathrm{mm}}^{3}}\right]=\frac{W_{2}-W_{1}}{\mathrm{specimen}\;\mathrm{volume}}$$

(2) $$\mathrm{Solubility}\;\left[\mu g/{\mathrm{mm}}^{3}\right]\;=\frac{W_{1}-W_{3}}{\mathrm{specimen}\;\mathrm{volume}}.$$

Statistical analysis was performed with repeated-measures one-way ANOVA for fluoride ion release and recharge with time, and one-way ANOVA for independent variables followed by Tukey’s HSD multiple comparison post hoc tests. Differences with *p* < 0.05 were considered statistically significant.

## 3. Results

**Figure 2:**
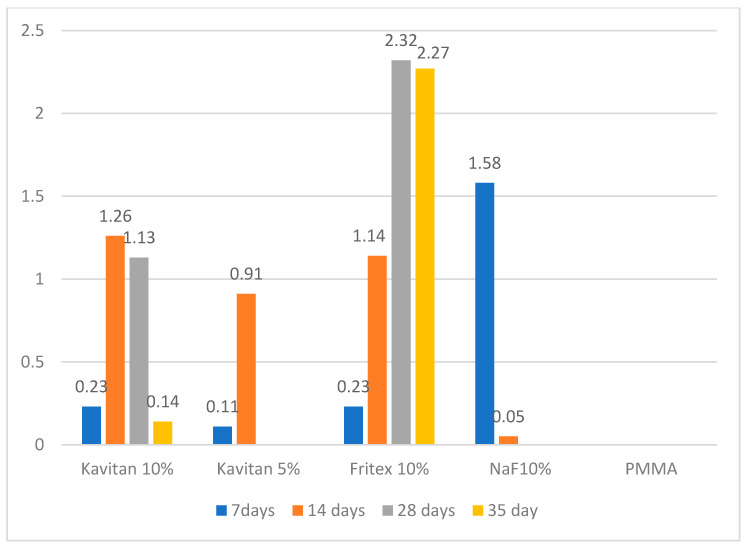
Fluoride release with time of storage.

**Figure 3:**
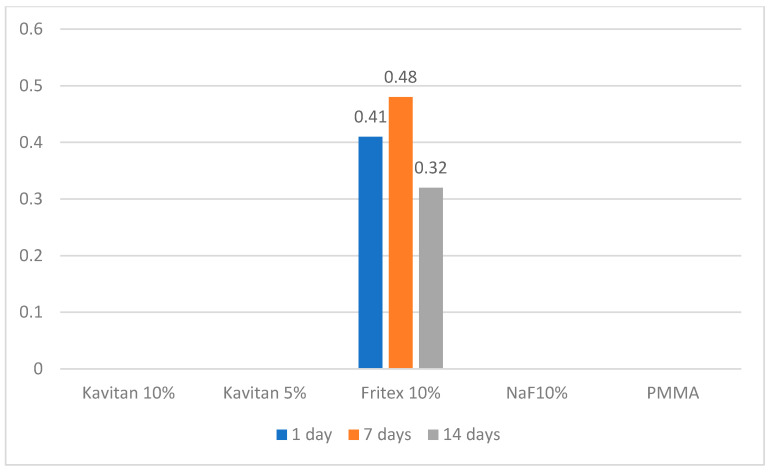
Amount of fluoride released after contact with the 1450 ppm fluoride toothpaste.

Table 3 shows significant differences between the materials and time points. Compared to the unmodified PMMA resin, all the tested modified materials could release fluoride ions during the first 14 days of the experiment. After 14 days, the only specimens still releasing fluoride ions were 10 wt% Kavitan and 10 wt% Fritex. For 10 wt% NaF-modified specimens, the highest fluoride ion release was noted after the 7th day of the experiment (1.58 µg/cm^2^). After 14 days, a rapid decrease in the amount of the released ions was noted. Ion release from 5 wt% Kavitan-modified specimen was the lowest of all the modified materials after 7 days of storage in distilled water. After 14 days, the release was higher than that for 10 wt% NaF-containing specimens but still lower than those noted for 10 wt% Kavitan- and 10 wt% Fritex-modified specimens. PMMA specimens modified with 10 wt% Kavitan glass released fluoride ions at the same level as that of specimens modified with 10 wt% Fritex glass (0.23 µg/cm^2^) after 7 days and had the highest ion release of all the tested materials after 14 days (1.26 µg/cm^2^). After 28 days, the only materials still releasing fluoride ions were those modified with 10 wt% Kavitan and 10 wt% Fritex, but for 10 wt% Fritex-modified specimens, the values increased and were the highest (2.32 µg/cm^2^) of all materials and remained at this level until the 35th day of the experiment, where no significant decrease was noted (Figure 2). Table 4 presents results obtained for experimental materials after recharge procedure with significant differences between the materials and time points. PMMA modified with 10 wt% Fritex glass was the only material to absorb fluoride ions from the solution and then effectively release them. The amount of released ions ranged from 0.32 to 0.48 µg/cm^2^. This range was constant for the first 7 days of storage. A decrease in ion release was observed between the 7th and 14th day of the experiment (Figure 3).

Sorption values ranged from 10.23 μm/mm^3^ for unmodified PMMA resin to 12.11 μm/mm^3^ for specimens modified with 10 wt% Kavitan glass. Materials modified with 10 wt% NaF as well as modified with 10 wt% Kavitan had higher sorption levels after 7 days 11.95 ± 0.33 μm/mm^3^ and 12.11 ± 0.8 μm/mm^3^ than the other tested materials and unmodified PMMA resin. Solubility of all tested materials ranged from 1.05 to 1.59 μm/mm^3^. No significant differences were found regarding solubility levels after 7 days (Table 5).

## 4. Discussion

The null hypothesis assumed for this study was rejected. Acrylic resin could release fluoride ions for a varying amount of time after modification with NaF or glasses from GIC. For the NaF filler, this period was very short, approximately 7 days, but the amount of released ions was very high. This rapid release may be explained by the high solubility of NaF and lack of its chemical bond with the acrylic resin. For specimens modified with 5 wt% and 10 wt% Kavitan glass, the amount of released ions systematically decreased after 14 days of storage in distilled water, and no recharge abilities were observed. For specimens modified with 10 wt% Fritex glass, the increase in released ions was observed until the 28th day of the experiment without a significant decrease on the 35th day. It was also the only tested material with fluoride recharge abilities. Sorption and solubility levels all tested materials confirmed with ISO 20795-1:2013 requirements for denture base materials and were below 32 μm/mm^3^ for sorption and 1.6 μm/mm^3^ for solubility.

The spectrophotometric method used in this study is a simple and cost effective alternative method for determining fluoride anions release form dental materials. The results are comparable with those obtained with use of an ion selective electrode reported in the studies of fluoride release from modified PMMA material [25,26]. Fluoride ions influence human population health. Fluoride releasing abilities are desirable for bioactive dental materials. The amount of released anions have to be on appropriate level to ensure protective action and also prevent its excessive intake, which can have adverse effect on human health [30,32]. An important question is what is the minimum concentration of fluoride that may significantly reduce the demineralization process and formation of caries lesions. Margolis et al. reported that enamel remineralization starts even with low fluoride levels ranging from 0.024 to 0.154 ppm [1,24]. Other studies concluded that a constant supply of low levels of fluoride ranging from 0.03 to 0.3 ppm for a prolonged period of time is most beneficial for caries control [18,33,34]. Hence, the continuous release of small amounts of fluoride ions from the denture base material in constant contact with the remaining dentition is favorable. Dedicated denture cleaning toothpastes containing fluoride in their composition may be a good daily source of fluoride ion recharge. Leaving the denture with the toothpaste slurry in water overnight may be an easy and economical procedure similar to “toothpaste technique” suggested by Sjögren [35]. Probably two methods of remineralization of the rest dentition could be useful, fluoride containing mouth hygiene products and fluoride releasing dental base resins. This combined application of fluoride might be a basis to design more effective methods and provide enhanced prevention and remineralization of dental enamel [6].

In the present study, the amount of released fluoride ions from specimens modified with 10 wt% Fritex glass after the recharge was over 10 times greater than the required minimum. It should also be noted that the specimens of the material used for the tests were very small (10 mm diameter, 1 mm thickness), which is approximately 50 times smaller than the average surface of the denture plate.

The results of the present study agree with those of Al-Bakri et al. [26]. The difference, however, is that in the present study, the filler was not a silane-modified glass, but only mechanically mixed with acrylic powder. Therefore, initially, the amount of released fluoride after 7 days was lower than that reported by Al-Bakri et al. (approximately 1 µg/cm^2^ reported by Al-Bakri et al. compared to 0.23 µg/cm^2^ for 10 wt% Kavitan- and 10 wt% Fritex-modified specimens), but after the 14th day, the ion release was at the similar level (0.9 µg/cm^2^ in the study of Al-Bakri et al. versus 1.26 µg/cm^2^ in the present experiment). A comparison between the glass filler GM35429 (Shofu) used by these authors and Kavitan and Fritex is that the Shofu glass contains higher concentration of fluoride ions (15%) than Kavitan (10%) and Fritex (6%). It is worth noting that the 10% concentration of glass in acrylic resin was effective in both studies, which is important regarding the flexural strength decrease associated with the increase in filler concentration [25,26]. Additionally, for it was reported in the study of Al-Bakri that despite the increase in roughness of the specimens modified with 10% fluoridated glass filler the least microbial adhesion was noted regarding Candida albicans and Streptococcus mutans. The decreased candidal and bacterial adhesion was explained by the constant fluoride releasing effect of the modified PMMA resin [27].

The amount of released fluoride ions and time duration of the release depends on the concentration of bioactive glass in the resin, which was observed for specimens containing 5 wt% and 10 wt% Kavitan. The most promising aspect from a clinical point of view is the release of fluoride ions from acrylic specimens containing 10 wt% Fritex glass. This material, as the only one among the tested materials, could re-release fluoride ions after an overnight contact with a toothpaste solution and then release them again into the oral cavity. The recharge ability of the tested materials might vary with use of different forms or concentrations of fluoridated products during recharge process. But even with the use of the simple and low-cost “toothpaste technique” recharge ability was noticed for this glass filler.

NaF at a concentration of 10% was also tested as an additive. This substance, which dissolves in a short period of time (7 days: 1.581 µg/cm^2^), releases a large amount of fluoride ions from its structure. However, the amount of released ions decreases significantly with time (after 14 days: 0.05 µg/cm^2^). This finding is in line with the study of Agarwal et al. for PMMA with a 20% addition of NaF. The authors of this study observed that after 3 days, the amount of fluoride release was 2 times lower than after the first hour [36]. This may be because NaF dissolves easily in water and is not chemically bonded with PMMA. Thus, even a short-term contact with water causes rapid washing out of this filler from acrylic resin. Resins modified with NaF and 2-hydroxyethyl methacrylate (HEMA) can release ions up to 28 days, as reported by Sabir et al. [37]. However, this was not confirmed in the present study, where fluoride ions could be detected in distilled water up to 7 days. This may be due to the different preparation methods of acrylic samples without covering NaF with HEMA and the dispersion of NaF in them.

The biocompatibility of fluoride-releasing acrylic resins was tested by Benton et al. using subcutaneous implantation methods on a guinea pig animal model. An unmodified acrylic resin was used as a reference material. The conclusions from this study indicate that fluoride-PMMA composition produces a very mild subcutaneous tissue response and that its biocompatibility is comparable to that of widely used nonmodified dental resins [38].

The limitation of the present study was that it was conducted using only distilled water as a storage medium. The amount of released fluoride ions may be higher in more acidic environment such as environment rich in lactic acid. Also, the physical and mechanical properties of the modified PMMA resins should be evaluated, because of possible alteration of the modified materials properties, such as increase of porosity, changes in hardness, elastic modulus and flexural strength. The in vitro study cannot fully mimic the oral cavity environment. In future studies, more bioactive fillers in various concentrations should be tested and the mechanical properties of the modified resins should be evaluated. The possible antifungal and antibacterial action of the proposed modified PMMA resins should also be investigated.

## 5. Conclusions

The addition of 10 wt% Fritex and 10 wt% Kavitan bioactive glass to heat-cured acrylic resin may improve the material properties of PMMA, with bioactive fluoride ion release ability lasting for over 4 weeks. The resin modified with 10 wt% Fritex glass could absorb fluoride ions from the toothpaste solution and then effectively release them. Addition of fluoride releasing fillers have a small effect on sorption and solubility increase of the modified PMMA resin.

## 6. Clinical Significance

The addition of bioactive glass may be promising in the development of the novel bioactive heat-cured denture base resin.

## Figures and Tables

**Table 1:** Compositions of glasses used in polymethyl methacrylate (PMMA) modification.

Kavitan Glass	Fritex
SiO_2_ (26%)	SiO_2_ (44%)
Al_2_O_3_ (29%)	Al_2_O_3_ 30%
F (10%)	F (6%)
P_2_O_5_ (8%)	P_2_O_5_ (3%)
Na_2_O (4%)	Na_2_O (6.0%)
SrO (17%)	CaO (5.0%)

**Table 2:** Specimen composition.

Group	Quantity	Composition
P0	10	Superacryl Plus (PMMA)
P1	10	Superacryl Plus + 5 wt% Kavitan Plus glass
P2	10	Superacryl Plus + 10 wt% Kavitan Plus glass
P3	10	Superacryl Plus + 10 wt% sodium fluoride
P4	10	Superacryl Plus + 10 wt% Fritex glass

**Table 3:** Mean values ± SD of fluoride release [µg/cm^2^].

	Time			
Material	7 Days	14 Days	28 Days	35 Days
PMMA	nd	nd	nd	nd
PMMA + NaF	1.58 ± 0.01 A a	0.05 ± 0.02 A b	nd	nd
PMMA + Kavitan 5 wt%	0.11 ± 0.01 B a	0.91 ± 0.05 B b	nd	nd
PMMA + Kavitan 10 wt%	0.23 ± 0.02 C a	1.26 ± 0.02 C b	1.13 ± 0.15 A b	0.14 ± 0.05 A a
PMMA + Fritex 10 wt%	0.23 ± 0.04 C a	1.14 ± 0.02 D b	2.32 ± 0.07 B c	2.27 ± 0.06 B c

nd—nondetectable. Small letters in rows and big letters in columns denote statistically significant differences with *p* ≤ 0.05.

**Table 4:** Mean values ± SD of fluoride release after recharge (µg/cm^2^).

Material	1 Days	7 Days	14 Days
PMMA	nd	nd	nd
PMMA + NaF	nd	nd	nd
PMMA + Kavitan 5 wt%	nd	nd	nd
PMMA + Kavitan 10 wt%	nd	nd	nd
PMMA + Fritex 10 wt%	0.41 ± 0.01 A a	0.48 ± 0.04 A a	0.32 ± 0.05 A b

nd—nondetectable. Small letters in rows and big letters in columns denote statistically significant differences with *p* ≤ 0.05.

**Table 5:** Mean values ± SD of sorption and solubility after 7 days (μm/mm^3^).

Material	Sorption	Solubility
PMMA	10.23 ± 0.63	1.05 ± 0.11
PMMA + NaF	11.95 ± 0.33 *	1.73 ± 0.15
PMMA + Kavitan 5 wt%	11.55 ± 1.25	1.51 ± 0.09
PMMA + Kavitan 10 wt%	12.11 ± 0.8 *	1.59 ± 0.05
PMMA + Fritex 10 wt%	11.61 ± 0.07	1.19 ± 0.05

* statistically significant differences with *p* ≤ 0.05.

## Data Availability

The data presented in this study are available on request from the corresponding author.

## References

[1] Margolis H., Moreno E., Murphy B. (1986). Effect of Low Levels of Fluoride in Solution on Enamel Demineralization in vitro. J. Dent. Res..

[2] Nakornchai N., Arksornnukit M., Kamonkhantikul K., Takahashi H. (2016). The pH effect of solvent in silanization on fluoride re-leased and mechanical properties of heat-cured acrylic resin containing fluoride-releasing filler. Dent. Mater. J..

[3] Featherstone J.D., Singh S., Curtis D.A. (2011). Caries risk assessment and management for the prosthodontic patient. J. Prosthodont..

[4] Burnett G., Nehme M., Parkinson C., Karwal R., Badrock T., Thomas G.V., Hall P. (2020). A randomised oral fluoride retention study comparing intra-oral kinetics of fluoride-containing dentifrices before and after dietary acid exposure. Arch. Oral Biol..

[5] Ullah R., Zafar M.S., Al-Munawwarah A.M. (2015). Oral and dental delivery of fluoride: A review. Fluoride.

[6] Byeon S.M., Lee M.H., Bae T.S. (2016). The effect of different fluoride application methods on the remineralization of initial carious lesions. Restor. Dent. Endod..

[7] Zafar M.S., Al-Madinah N.A., Al-Munawwarah A.M. (2015). Therapeutic roles of fluoride released from restorative dental materials. Fluoride.

[8] Domagała I., Przystupa K., Firlej M., Pieniak D., Niewczas A., Biedziak B. (2020). Bending Behaviour of Polymeric Materials Used on Biomechanics Orthodontic Ap-pliances. Materials.

[9] Zafar M.S. (2020). Prosthodontic Applications of Polymethyl Methacrylate (PMMA): An Update. Polymers.

[10] Cochrane N., Cai F., Huq N., Burrow M., Reynolds E. (2010). New Approaches to Enhanced Remineralization of Tooth Enamel. J. Dent. Res..

[11] Verma A., Khurshid S., Parveen F., Khanna S., Pandey P. (2015). Remineralization: An approach towards conservation of tooth. J. Evol. Med. Dent. Sci..

[12] Van Loveren C. (2001). Antimicrobial activity of fluoride and its in vivo importance: Identification of research questions. Caries Res..

[13] Flisfisch S., Meyer J., Meurman J., Waltimo T., Meurman J. (2008). Effects of fluorides on Candida albicans. Oral Dis..

[14] Naoum S., Ellakwa A., Martin F., Swain M. (2011). Fluoride release, recharge and mechanical property stability of various fluo-ride-containing resin composites. Oper. Dent..

[15] Harhash A.Y., El Sayad I.I., Zaghloul A.G.S. (2017). A comparative in vitro study on fluoride release and water sorption of different flowable esthetic restorative materials. Eur. J. Dent..

[16] Mitwalli H., Balhaddad A.A., AlSahafi R., Oates T.W., Melo M.A.S., Xu H.H.K., Weir M.D. (2020). Novel CaF2 Nanocomposites with Anti-bacterial Function and Fluoride and Calcium Ion Release to Inhibit Oral Biofilm and Protect Teeth. J. Funct. Biomater..

[17] Sidhu S.K., Nicholson J.W. (2016). A Review of Glass-Ionomer Cements for Clinical Dentistry. J. Funct. Biomater..

[18] Kiatsirirote K., Sitthisettapong T., Phantumvanit P., Chan D.C.N. (2019). Fluoride-Releasing Effect of a Modified Resin Denture Con-taining S-PRG Fillers on Salivary Fluoride Retention: A Randomized Clinical Study. Caries Res..

[19] Fujimoto Y., Iwasa M., Murayama R., Miyazaki M., Nagafuji A., Nakatsuka T. (2010). Detection of ions released from S-PRG fillers and their modulation effect. Dent. Mater. J..

[20] Mousavinasab S.M., Meyers I. (2009). Fluoride Release by Glass Ionomer Cements, Compomer and Giomer. Dent. Res. J..

[21] Muñoz C.R.D., Ramírez Ortega J.P., Yamamoto Nagano A. (2014). Fluoride release of two glass-ionomer cements: In vitro study. Revista Odontológica Mexicana.

[22] Basso G.R., Della Bona A., Gobbi D.L., Cecchetti D. (2011). Fluoride release from restorative materials. Braz. Dent. J..

[23] Arbabzadeh-Zavareh F., Gibbs T., Meyers I.A., Bouzari M., Mortazavi S., Walsh L.J. (2012). Recharge pattern of contemporary glass ionomer restoratives. Dent. Res. J..

[24] Mukai Y., Kamijo K., Fujino F., Hirata Y., Teranaka T., Cate J.M.T. (2009). Effect of denture base-resin with prereacted glass-ionomer filler on dentin demineralization. Eur. J. Oral Sci..

[25] Kamijo K., Mukai Y., Tominaga T., Iwaya I., Fujino F., Hirata Y., Teranaka T. (2009). Fluoride release and recharge characteristics of denture base resins containing surface pre-reacted glass-ionomer filler. Dent. Mater. J..

[26] Al-Bakri I.A., Swain M.V., Naoum S.J., Al-Omari W.M., Martin E., Ellakwa A. (2014). Fluoride release, recharge and flexural properties of polymethylmethacrylate containing fluoridated glass fillers. Aust. Dent. J..

[27] Al-Bakri I.A., Harty D., Al-Omari W.M., Swain M.V., Chrzanowski W., Ellakwa A. (2014). Surface characteristics and microbial adher-ence ability of modified polymethylmethacrylate by fluoridated glass fillers. Aust. Dent. J..

[28] Tsutsumi C., Takakuda K., Wakabayashi N. (2016). Reduction of Candida biofilm adhesion by incorporation of prereacted glass ionomer filler in denture base resin. J. Dent..

[29] Tamura M., Cueno M.E., Abe K., Kamio N., Ochiai K., Imai K. (2018). Ions released from a S-PRG filler induces oxidative stress in Candida albicans inhibiting its growth and pathogenicity. Cell Stress Chaperones.

[30] Ünal E.I., Kenar A., Aksu M.L., Taştekin M. (2019). Spectrophotometric methods for the determination of fluoride ion using in-dole-3-acetic acid interaction with iron (III). Turk. J. Chem..

[31] ISO (2013). ISO 20795-1:2013 Dentistry—Base Polymers—Part 1: Denture Base Polymers.

[32] Kamel R.M., Shahat A., Hegazy W.H., Khodier E.M., Awual R. (2019). Efficient toxic nitrite monitoring and removal from aqueous media with ligand based conjugate materials. J. Mol. Liq..

[33] Sjögren K., Birkhed D. (1993). Factors Related to Fluoride Retention after Toothbrushing and Possible Connection to Caries Activity. Caries Res..

[34] Duckworth R., Morgan S. (1991). Oral Fluoride Retention after Use of Fluoride Dentifrices. Caries Res..

[35] Sjögren K. (1995). Toothpaste technique. Studies on fluoride delivery and caries prevention. Swed. Dent. J Suppl..

[36] Agarwal B., Dayal Singh R., Raghav D., Shekhar A., Yadav P. (2019). Determination of Fluoride Release and Strength of a Fluoride Treated Heat Cured Acrylic Resin. EAS J. Dent. Oral Med..

[37] Sabir D., Omer Z. (2019). Evaluation of Fluoride release from orthodontic acrylic resin by using two different polymerizations techniques: An In Vitro Study. Erbil Dent. J. (EDJ).

[38] Benton J.B., Zimmerman B.F., Zimmerman K.L., Rawls H.R. (1993). In vivo biocompatibility of an acrylic, fluoride-releasing, ani-on-exchange resin. J. Appl. Biomater. Off. J. Soc. Biomater..

